# Maintenance of complete mucosal healing is associated with avoiding restenosis after endoscopic balloon dilation of Crohn's disease‐related small intestinal strictures

**DOI:** 10.1002/deo2.239

**Published:** 2023-04-17

**Authors:** Ulzii Dashnyam, Manabu Nagayama, Tomonori Yano, Hirotsugu Sakamoto, Makiko Mieno, Jun Owada, Kunihiko Oguro, Tsevelnorov Khurelbaatar, Keijiro Sunada, Alan Kawarai Lefor, Hironori Yamamoto

**Affiliations:** ^1^ Department of Medicine Division of Gastroenterology Jichi Medical University Tochigi Japan; ^2^ Department of Medical Informatics Center for Information Jichi Medical University Tochigi Japan; ^3^ Department of Surgery Jichi Medical University Tochigi Japan; ^4^ Department of Pediatrics Mongolian National University of Medical Sciences Ulaanbaatar Mongolia; ^5^ Endoscopy Center, Mongolian Japan Hospital Mongolian National University of Medical Sciences Mongolia Ulaanbaatar

**Keywords:** calibrated small‐caliber‐tip transparent hood, Crohn's disease, double‐balloon enteroscopy, endoscopic balloon dilation, endoscopic restenosis

## Abstract

**Background:**

Endoscopic balloon dilation (EBD) is an effective, minimally invasive treatment for Crohn's disease (CD) related intestinal strictures. However, restenosis frequently occurs and requires repetitive EBD or surgical resection. Since previous studies could not evaluate restenosis based on stricture diameter, factors affecting restenosis after EBD were unclear. This study aimed to identify these factors by precisely measuring the diameter of small intestinal strictures in patients with CD.

**Methods:**

This single‐center retrospective study enrolled patients with CD with de novo small intestinal strictures who underwent two double‐balloon enteroscopy sessions (EBD and follow‐up) between January 2016 and October 2021. Clinical and endoscopic data were obtained from electronic medical records. A calibrated small‐caliber‐tip transparent hood was used to precisely measure stricture diameters. Multivariate analysis was performed to identify factors associated with restenosis.

**Results:**

Forty‐eight patients (37 male) were analyzed. The total number of strictures detected decreased from 162 to 143. The mean diameter of all strictures and the narrowest stricture in each patient increased significantly from 8.6 to 9.8 mm and from 7.6 to 8.7 mm, respectively. Thirty‐two (67%) patients developed endoscopic restenosis. Multivariate analysis showed that the presence of ulcers at the follow‐up session was a risk factor for restenosis (odds ratio 9.4, *p* = 0.01). Patients with complete mucosal healing at both sessions (*n* = 21) showed significant improvement in the narrowest stricture (+1.7 mm, *p* = 0.001).

**Conclusions:**

Maintenance of complete mucosal healing is significantly associated with avoiding restenosis after EBD in CD‐related small intestinal strictures.

## INTRODUCTION

Crohn's disease (CD) is a phenotype of inflammatory bowel disease characterized by repeated episodes of clinical remission and relapse. CD‐specific lesions can lead to fibrotic intestinal strictures, which are the most common complication of CD.^1^, ^2^ The cumulative rate of stricture formation increases from 11%–14% to 21%–35% 5 years after the initial diagnosis despite recent advances in medical treatment.^3^, ^4^ Symptomatic small intestinal strictures are the most common indication for CD‐associated surgery.^4^ If patients underwent surgical resection of strictures, subsequent operations are often required, with a 5‐year risk for a second operation of 24.2%.^5^ Repeated surgical resections can lead to short bowel syndrome; therefore, endoscopic balloon dilation (EBD) is becoming a favored alternative to surgery.^6^

EBD is less invasive and relatively safe and effective for uncomplicated and short strictures (<5 cm).^7^, ^8^ However, restenosis after EBD often occurs, requiring repeat EBD or surgical intervention.^9^ Previous studies evaluating the long‐term efficacy of EBD used symptom‐free rates and surgery‐free rates as evidence of an absence of restenosis.^10^, ^11^, ^12^ However, obstructive symptoms can be influenced by eating habits, and the decision to perform surgery depends on the patient's and physician's intentions. Therefore, objective evaluation of strictures is important for effective management. Objective measurement of the inner diameter of strictures is not widely performed but is performed at our institution during scheduled double‐balloon enteroscopy (DBE) sessions. For this reason, we aimed to assess the effectiveness of EBD, detect the restenosis rate and identify factors causing restenosis.

## PATIENTS/MATERIAL AND METHODS

### Patients

Annually scheduled DBE is performed for most patients with CD to monitor and optimize medications in our institution. When a stricture is found during the DBE, EBD is performed to pass through the stricture and observe further areas. This retrospective cohort study initially reviewed data from 113 patients with CD who underwent two serial sessions of scheduled DBE with EBD between January 2016 and October 2021. Patients who met the following inclusion criteria were then selected: (1) patients with ileal or ileocolonic CD^13^, (2) patients with only de novo stricture(s) in the small bowel, which met the indications for EBD, (3) all strictures were reachable and underwent EBD, and (4) patients underwent two DBE sessions with an interval of 7–24 months (1st session: EBD, 2nd session; follow‐up). EBD was indicated for short (<5 cm) fibrous strictures through which the endoscope or over‐tube could not be passed regardless of obstructive symptoms and was not indicated for strictures with deep ulcers, fistulas, severe angulations, and abscesses. Finally, patients with insufficient data were excluded. The study was approved by the Institutional Ethical Review Board of Jichi Medical University and conducted in accordance with the principles of the Declaration of Helsinki.

### Sessions and procedures

A series of DBE during one hospitalization was considered “one session of DBE” in this study. At the EBD session and the follow‐up session, all detected strictures were evaluated and treated by EBD as indicated. Patients gave written informed consent for DBE, EBD, and conscious sedation before the procedures.

***DBE***: The presence of ulcers and strictures were evaluated by therapeutic‐type DBE (EN‐580T or EI‐580BT; Fujifilm Corporation, Tokyo, Japan) with an over‐tube (TS‐13140 or TS‐13101; Fujifilm Corporation). An ulcer was defined as a mucosal defect, not depending on size or depth, and includes very tiny mucosal defects. A stricture was defined as a narrowed lumen with an inner diameter of 13 mm or less than the endoscope or over‐tube that could not pass through at the EBD session.

**FIGURE 1:**
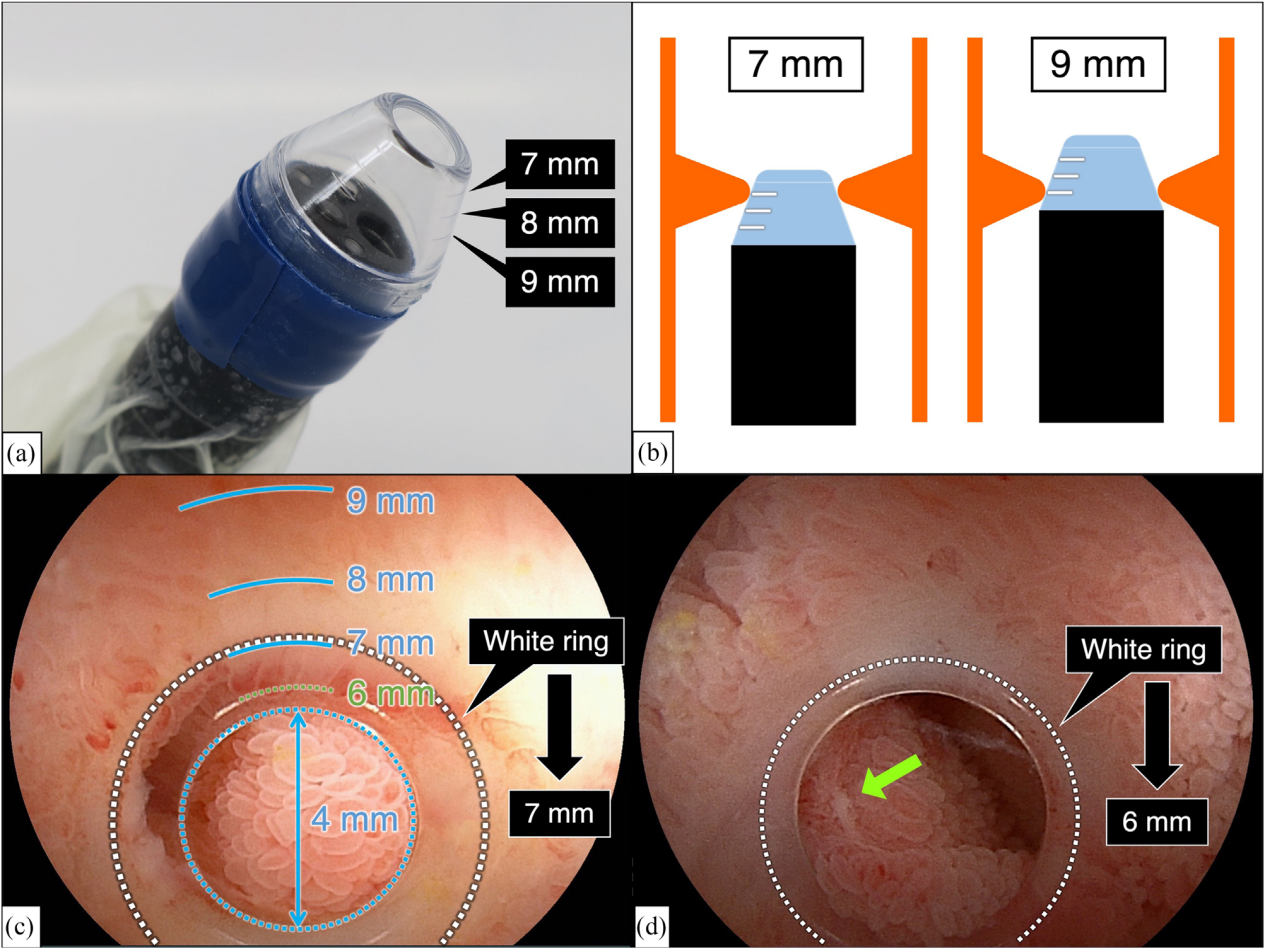
Calibrated small‐caliber‐tip transparent (CAST) hood and representative pictures of small intestine strictures. (a) A CAST hood attached to the tip of the endoscope. (b) Measurement of the inner diameter by wedging the CAST hood in the stricture. (c) Calibration lines on the wall of the CAST hood are highlighted in blue. The white ring indicates the narrowest part of the stricture. The inner diameter is read as 7 mm. (d) Severe stricture (6 mm) with a small ulcer (arrow).

***Measuring stricture diameter***: Stricture diameters were measured by using a calibrated small‐caliber‐tip transparent hood (CAST hood, Top Corporation, Tokyo, Japan; Figure 1a,b), which was invented for sequential EBD and measuring the diameter of a stricture (Figure 1c,d).^14^, ^15^ Calibration lines (7, 8, and 9 mm) and other landmarks (orifice: 4 mm, edge: 6 mm, outer ring: 10 mm) on the CAST hood allow objective measurement by identifying “a white ring”, which is seen during cautious insertion into the stricture and indicates the fibrous part of the stricture (Figure 1c).^14^, ^15^ Diameters of mild strictures were estimated from the resistance of the CAST hood attached scope and over‐tube with outer diameters of 11 mm and 13.2 mm, respectively, to pass through the stricture (Table 1). Based on the diameter, all detected strictures were classified as mild (diameter ≥11 mm), moderate (diameter 8–10 mm), and severe (diameter ≤ 7 mm).

**TABLE 1:** Methods to measure the diameter of strictures using the calibrated small‐caliber‐tip transparent hood.

Diameter of stricture	Methods to describe the diameter of strictures
4 mm	The white ring is the same as the opening of the CAST hood
5 mm	The white ring is larger than the opening but less than the edge of the CAST hood
6 mm	The white ring is the same as the edge of the CAST hood
7–9 mm	The calibration lines of the CAST hood at the white ring
10 mm	Scope passing with a certain resistance
11 mm	Scope passing with slight resistance
12 mm	Scope passing without resistance.
13 mm	Over‐tube passing with a certain resistance
14 mm	Over‐tube passing with slight resistance
15 mm	Over‐tube passing without resistance

Abbreviation: CAST, calibrated small‐caliber‐tip transparent.

***EBD***: After measurement, an endoscopic guidewire (Revowave, Piolax, Kanagawa, Japan) and the through‐the‐scope balloon catheter (CRE balloon catheter, Boston Scientific, MA, USA) were inserted through the stricture lumen and inflated up to the target diameter under fluoroscopic guidance. The dilating balloon was then pressurized for one minute. Other subsequent strictures were measured and treated with EBD in patients with multiple strictures. Although EBD for mild strictures was not necessary to pass the endoscope, it was often performed to pass the over‐tube depending on needs. A 12–15 mm balloon catheter was generally used for moderate and mild strictures. A 10–12 mm or 8–10 mm balloon catheter was mainly used for severe strictures. The target diameter of EBD was adjusted considering the balance of risks and benefits based on various factors, including the presence of ulceration, the diameter and length of the stricture, and the necessity of passing the scope or the overtube.

### Outcomes analysis

Endoscopic findings, including the frequency and diameters of strictures at both sessions, were compared to assess the effect of one EBD session. Since most patients had multiple strictures and some strictures became unrecognizable at the follow‐up session, we selected the diameter of the narrowest stricture in each session from each patient to define restenosis and improvement of stricture after EBD. In accordance with this definition, improvement was defined as a change of +2 mm or more in the diameter of the narrowest strictures, whereas restenosis was defined as a change of +1 mm or less. Patients were classified into improved and restenosis groups. Factors associated with restenosis after EBD were then analyzed.

### Statistical analysis

All statistical analyses were performed using STATA 16.1 (StataCorp, College Station, TX, USA). Fisher's exact test was used for group comparison of categorical variables. Student's t‐test or a paired t‐test was used to compare quantitative variables. Logistic regression analysis was used to identify factors associated with restenosis. Variables with a *p*‐value < 0.1 in univariate analysis were included in the multivariate analysis. A *p‐*value < 0.05 was considered statistically significant.

## RESULTS

### Background characteristics of patients

**FIGURE 2:**
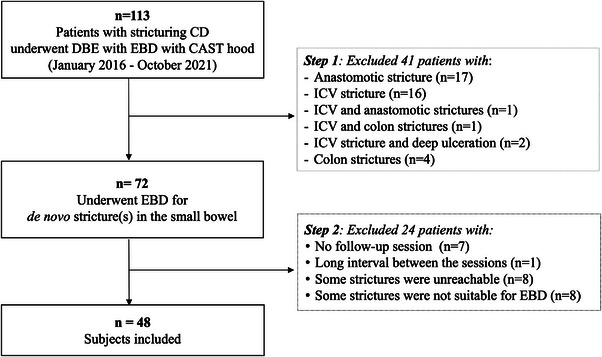
Study flowchart. Among 113 screened patients, 65 were excluded from the study after two exclusion steps, and 48 were finally included in the study. CAST hood, calibrated small‐caliber‐tip transparent hood; CD, Crohn's disease; DBE, double‐balloon enteroscopy; EBD, endoscopic balloon dilation; ICV, ileocecal valve.

**FIGURE 3:**
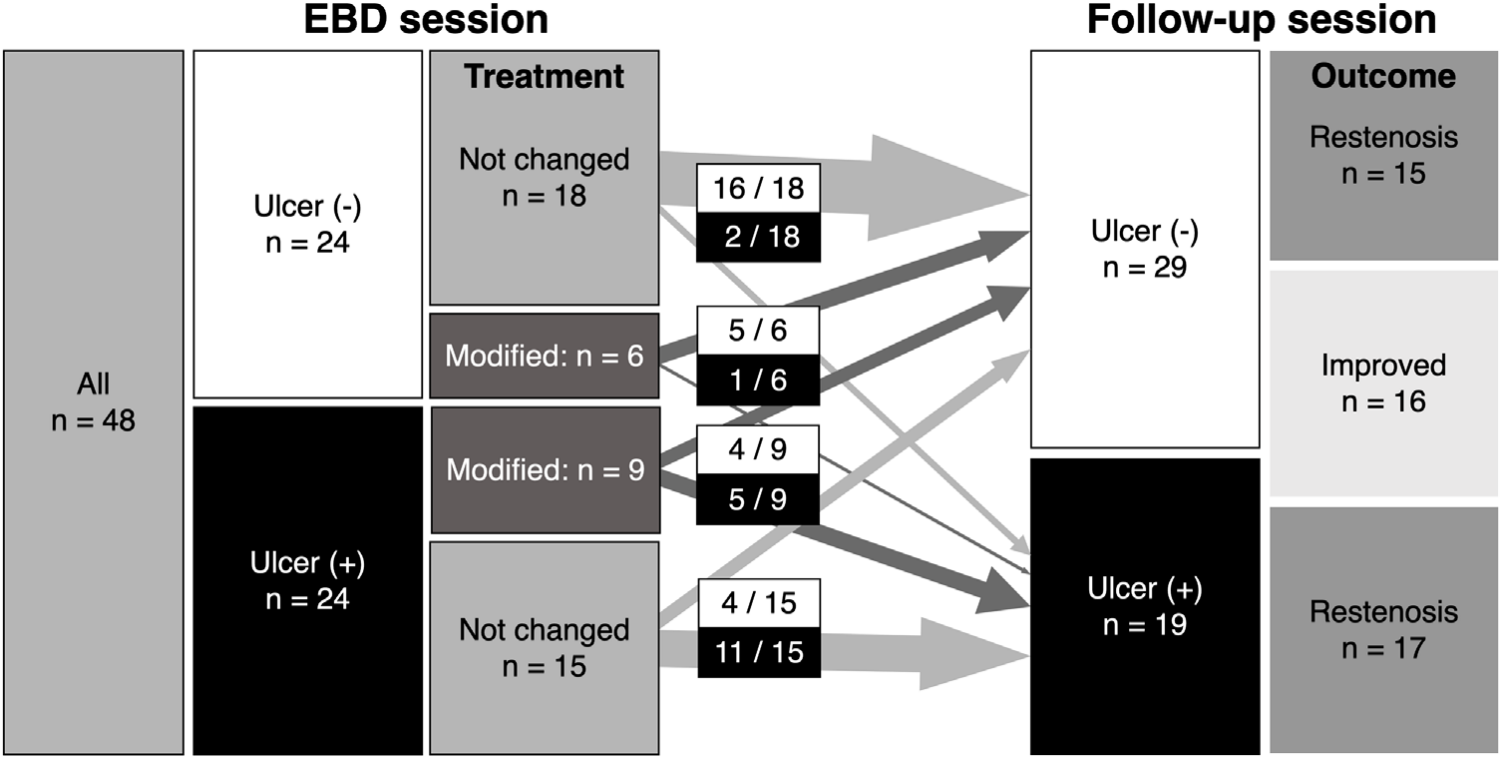
Treatment modification and clinical course after the endoscopic balloon dilation (EBD) session.

Among 113 patients, 65 patients were excluded, and 48 (37 male) were finally analyzed in this study (Figure 2). General characteristics and clinical findings of the patients are shown in Table 2. Seventy‐five percent (*n* = 36) of patients underwent the follow‐up session 10–14 months after the EBD session, and the mean interval between the sessions was 12.8 months. Most patients were asymptomatic and serologically in remission, with no significant differences in abdominal symptoms or laboratory data (Table 2) at the sessions. Patients underwent medical treatment based on evidence‐based clinical practice guidelines for inflammatory bowel disease in Japan^16^ at the time of the EBD session, with biologics (*n* = 34, 71%) and immunomodulators (*n* = 27, 56%). Medical treatment was modified in 15 patients (31%) after the EBD session due to the presence of ulcers and severe strictures, including the introduction of biologics (*n* = 5) and a dose escalation of immunomodulators and/or biologics (*n* = 7) (Table 3). Among patients with ulcers (*n* = 24), the ulcer healing rate tended to be higher in the group who underwent treatment modification (4/9) than in those who did not (4/15); Figure 3).

**TABLE 2:** Patient characteristics.

Characteristics	All *n* = 48	Restenosis *n* = 32	Improved *n* = 16	*p‐*Value
Gender (male: female)	37:11	24:8	13:3	0.73
Age (years), median (range)				
at diagnosis of Crohn's disease	30 (14–72)	32 (15–72)	28.5 (14–50)	0.46
at first EBD in the disease course	36.5 (17–74)	38 (17–74)	32 (18–50)	0.25
at the EBD session of the study	39.5 (17–81)	40.5 (17–81)	35 (18–53)	0.08
Age at diagnosis, *n* (%)				
A1: below 16 years	4 (8.3)	2 (6.3)	2 (13)	
A2: between 17 and 40 years	34 (71)	22 (69))	12 (75)	0.5
A3: above 40 years	10 (21)	8 (25)	2 (13)	
Overall period from initial diagnosis, (years), mean (±SD)				
to the first EBD of the disease course	4.5 ± 6.4	4.9 ± 7.0	3.6 ± 5.2	0.53
to the EBD session of the study	7.9 ± 7.1	9.3 ± 7.6	5.3 ± 5.1	0.07
History of EBD before the study, *n* (%)	30 (63)	24 (75)	6 (38)	0.01
Number of previous EBDs, mean (±SD)	3.1 ± 3.8	3.8 ± 3.7	1.8 ± 3.7	0.08
Period between the sessions, (months), mean (±SD)	12.8 ± 3.3	11.9 ± 2.6	14.7± 3.8	0.005
Patients with abdominal symptoms				
before EBD session, *n* (%)	11 (23)	7 (64)	4 (36)	0.1
before follow‐up session, *n* (%)	9 (19)	4 (44)	5 (56)	0.8
Laboratory data at EBD session, mean (±SD)				
Hemoglobin, g/dl	13.5 ± 1.4	13.4 ± 1.3	13.7 ± 1.4	0.5
Erythrocyte sedimentation rate, mm/h	8.5 ± 9.8	8.5 ±10.6	8.3 ± 8.2	0.94
C‐reactive protein, ssmg/dl	0.06 ± 0.1	0.07 ± 0.15	0.04 ± 0.05	0.48
Serum albumin, g/dl	4.4 ± 0.3	4.4 ± 0.3	4.5 ± 0.4	0.1
Laboratory data at follow‐up session, mean (±SD)				
Hemoglobin, g/dl	13.8 ± 1.3	13.7 ± 1.3	13.9 ± 1.2	0.52
Erythrocyte sedimentation rate, mm/h	6.4 ± 6.4	6.3 ± 7.1	6.7 ± 5.0	0.84
C‐reactive protein, mg/dl	0.09 ± 0.3	0.1 ± 0.32	0.08 ± 1.5	0.73
Serum albumin, g/dl	4.4 ± 0.3	4.4 ± 0.32	4.4 ± 0.34	0.38

Abbreviations: EBD; endoscopic balloon dilation; SD, standard deviation.

**TABLE 3:** Medical treatments before and after the endoscopic balloon dilation session.

Medical treatments	All	Restenosis *n* = 32	Improved *n* = 16	*p‐*Value
Before the EBD session, *n* (%)				
Elemental nutrition	43 (90)	28 (88)	15 (94)	0.5
5‐aminosalycilic acid	41 (85)	27 (84)	14 (88)	0.8
Systemic steroids	8 (17)	4 (6)	4 (25)	0.3
Immunomodulators	27 (56)	17 (53)	10 (63)	0.5
Biologics	34 (71)	22 (69)	12 (75)	0.7
After EBD session, *n* (%)				
Elemental nutrition	43 (90)	29 (91)	14 (88)	1.0
5‐aminosalycilic acid	40 (83)	26 (81)	14 (88)	0.7
Systemic steroids	6 (13)	2 (6.2)	4 (25)	0.09
Immunomodulators	27 (56)	17 (53)	10 (63)	0.76
Biologics	39 (81)	26 (81)	13 (81)	1.0
Treatment modified after EBD session, *n* (%)	15 (31)	10 (31)	5 (31)	1.0

Abbreviation: EBD; endoscopic balloon dilation.

### Procedure‐related adverse event

One hundred and twenty DBE procedures were performed during both sessions. Two episodes of post‐procedural bleeding were observed and treated conservatively. Perforation or acute pancreatitis was not observed.

### Outcomes of EBD

At the EBD session, 162 strictures were detected and dilated (≤13.5 mm for 104 and 15 mm for 58). At the follow‐up session, 143 strictures were detected, and 111 strictures were dilated. The mean number of detectable strictures per patient decreased from 3.4 to 3.0 (*p* = 0.04). The number of strictures decreased in 16 (33%) patients, six of whom had no stricture that warranted treatment. Patients with ulcerative strictures decreased from 24 (50%) to 19 (40 %). The frequency of severe and moderate strictures decreased from 57 to 40 and from 75 to 46, respectively. The mean diameter of detectable strictures increased significantly from 8.7 to 9.7 mm (*p* = 0.0004). The mean diameter of strictures in each patient increased in 33 (70%) patients, and the mean diameter of all strictures and the narrowest stricture in each patient improved significantly from 8.6 to 9.8 mm (*p* < 0.0001) and 7.6 to 8.7 mm (+1.1 mm, *p* = 0.0001).

Of the 48 study patients, 32 (67 %) patients had restenosis, and 16 (33%) were improved (Tables 2, 3, 4 and Figure 3). To identify factors associated with restenosis, univariate analysis was performed and revealed that a history of previous EBD, the interval between the two sessions, the use of a 15 mm dilation balloon, and the presence of ulcers at the follow‐up session were significantly associated with restenosis (Table 5). Multivariate analysis then showed the presence of ulcers at the follow‐up session was positively associated with restenosis (odds ratio 11.5; 95% CI 1.07–123.4, *p* = 0.03), and a long interval to follow‐up session after EBD and 15 mm sized dilation balloon were negatively associated with restenosis (odds ratio 0.7; 95% CI 0.46–0.98, *p* = 0.04 and odds ratio 0.1; 95% CI 0.01–0.64, *p* = 0.02; Table 5).

**TABLE 4:** Endoscopic findings in patients with restenosis and improvement after endoscopic balloon dilation.

Findings	All *n* = 48	Restenosis *n* = 32	Improved *n* = 16	*p‐*Value
Number of strictures per patient in the EBD session, *n*	3.4 ± 3.0	3.5 ± 3.3	3.1 ± 2.2	0.6
Presence of ulcer in the EBD session, *n*	24	18 (56)	6 (38)	0.4
Presence of ulcer in the follow‐up session, *n*	19	17 (53)	2 (13)	0.01
Mean diameter of all strictures in each patient in the EBD session, mm	8.6 ± 2.0	8.3 ± 1.9	9.2 ± 2.2	0.1
Diameter of the narrowest stricture in each patient in the EBD session, mm	7.6 ± 2.1	7.4 ± 1.9	8.1 ± 2.3	0.3
Mean diameter of dilation balloon for the narrowest strictures in the EBD session, mm	13.4 ± 1.6	12.9 ± 1.5	14.5 ± 0.9	0.0002

Data are presented as *n* (%) or mean ± standard deviation; EBD, endoscopic balloon dilation.

**TABLE 5:** Univariate and multivariate analysis of factors associated with restenosis.

Factors	Univariate analysis			Multivariate analysis		
	OR	95% CI	*p‐*Value	OR	95% CI	*p‐*Value
Gender (male)	0.69	0.16–3.06	0.63			
Age						
at diagnosis of Crohn's disease	1.02	0.97–1.07	0.45			
at first EBD during disease course	1.03	0.98–1.09	0.25			
at EBD session of the study	1.05	0.99–1.11	0.09	1.03	0.95–1.1	0.4
Overall period from initial diagnosis, years						
to first EBD during disease course	1.03	0.93–1.15	0.52			
to EBD session of the study	1.12	0.98–1.28	0.09	0.9	0.84–1.23	0.6
History of EBD before the study (>1 time)	5.0	1.37–18.1	0.01	3.6	0.32–39.8	0.3
Number of previous EBDs	1.2	0.97–1.46	0.09	1.1	0.81–1.6	0.5
Period between the sessions, months	0.7	0.57–0.95	0.02	0.7	0.46–0.98	0.04
Laboratory data in the EBD session						
Hemoglobin	0.97	0.94–1.02	0.3			
Erythrocyte sedimentation rate	0.97	0.93–1.02	0.3			
C‐reactive protein	2.99	0.04–25.3	0.62			
Serum albumin	0.93	0.81–1.06	0.92			
Treatment modification after EBD session (yes/no)*	1	0.27–3.63	1.0			
Concomitant treatments after EBD session (yes/no)*						
Elemental diet	0.93	0.16–6.1	1.0			
5‐aminosalycilic acid	0.61	0.11–3.5	0.59			
Systemic steroids	0.29	0.04–1.95	0.2			
Immunomodulators	0.68	0.19–2.32	0.53			
Biologics	0.82	0.18–3.7	0.8			
Endoscopic findings						
Multiple strictures in the EBD session (yes)*	1.62	0.2–6.2	0.48			
Number of strictures per patient in the EBD session	1.05	0.85–1.31	0.61			
Presence of an ulcer in the EBD session	2.14	0.62–7.3	0.22			
Presence of ulcer in the follow‐up session (yes/no)*	7.9	1.54–40.7	0.01	11.5	1.07–123.4	0.03
Diameter of stricture in the EBD session	0.85	0.63–1.14	0.28			
Presence of severe stricture in the EBD (diameter ≤ 7 mm) (yes/no)*	2.8	0.8–9.59	0.11			
Dilation balloon ≥ 15mm	0.14	0.04–0.53	0.004	0.1	0.01–0.64	0.02

Abbreviations: CI, confidence interval; EBD, endoscopic balloon dilation; OR; odds ratio.

* Reference = no

Patients with restenosis had a history of undergoing significantly more EBD procedures (3.8 vs. 1.8, *p* = 0.01). Of the narrowest strictures, 67 % (*n* = 32) were dilated by balloons 13.5 mm or less in diameter, and the remaining 33 % (*n* = 16) were treated with 15 mm balloons. Additionally, patients whose narrowest stricture was treated with 15 mm balloons tended to improve, whereas those treated with ≤13.5 mm balloons did not. Although there were no significant differences between the restenosis and improved patients, 69% of multiple stricture patients (25/36) and 74% of patients with severe strictures (20/27) developed restenosis.

**FIGURE 4:**
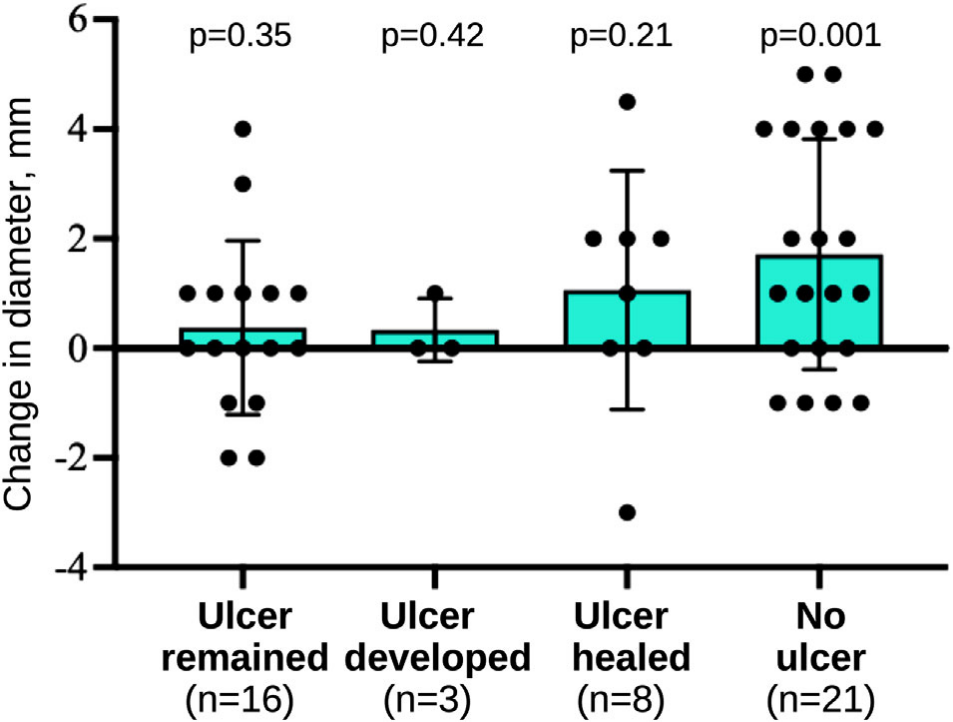
Changes in diameter of the narrowest strictures. Each dot represents a change in diameter in each patient. Bars and error bars represent mean and standard deviation, respectively. An asterisk indicates a *p*‐value < 0.05. Paired *t*‐test.

There were significantly more patients with ulcers at the follow‐up session in the restenosis group (53% vs. 13%, *p* = 0.01) compared to the improvement group. (Table 4). additionally, 75% (18/24) of patients with ulcers at the EBD session and 90% (17/19) of patients with ulcers at the follow‐up session developed restenosis. Since ulceration was associated with restenosis, we categorized patients into groups including ulcer‐remaining (*n* = 16), ulcer‐developed (*n* = 3), ulcer‐healed (*n* = 8), and no‐ulcer (*n* = 21; Figure 3) according to the presence of ulcers and sessions. Only the no‐ulcer group, which involved patients with complete mucosal healing throughout the study period, showed significant improvement in the mean diameter of the narrowest strictures (7.4 to 9.1, +1.7 mm, *p* = 0.001; Figure 4). The rates of patients who developed restenosis were highest among ulcer‐developed (100%, *n* = 3) and ulcer‐remaining (88%, *n* = 14) patients, while ulcer‐healed (50%, *n* = 4) and the no‐ulcer (52%, *n* = 11) groups had a lower rate.

## DISCUSSION

The present study identified factors associated with the recurrence of CD‐associated small intestinal strictures after dilation therapy by assessing the effect of one‐time EBD. Although the number of strictures decreased, and the diameters of the dilated strictures were significantly improved, restenosis also occurred in two‐thirds of patients in the present study. Analysis to identify factors associated with restenosis showed that patients with refractory and developed ulcerations at the follow‐up session had a higher risk of developing restenosis. Patients with complete mucosal healing had a significantly increased rate of improvement in the diameter of the narrowest stricture after EBD. A long interval between the two sessions and EBD up to 15 mm were associated with a decreased risk of restenosis.

EBD is widely used for CD‐related small intestinal strictures. Although a high rate of long‐term success has been demonstrated in numerous previous studies,^11^, ^17^, ^18^, ^19^, ^20^ rates of patients requiring frequent repeat dilations are still high. Restenosis was not reported with evidence of post‐EBD changes in the diameter of strictures because measuring the diameter of intestinal strictures accurately is difficult and not widely performed.

To the best of our knowledge, this is the first study to precisely evaluate the diameters of strictures using a CAST hood, which enables measuring the diameter of the stricture during DBE. As a result of the present study, a significant decrease in the frequency of strictures and an improvement in the diameter of strictures are demonstrated. For instance, all detected strictures significantly increased their mean diameter by 1 mm, and some strictures became unrecognizable due to an increase in diameter.

However, a high rate of repeat dilation due to restenosis after EBD was reported in many previous studies. This led to the creation of a new definition of restenosis in this study. Since 70% of patients in the present study had multiple strictures, and the number of detectable strictures was different at the two sessions, it was difficult to identify and compare each discrete stricture one‐to‐one in both sessions to define restenosis. Therefore, the change in diameter of only the narrowest stricture in each patient at the two sessions was considered. As a result, the restenosis rate was high (67%), similar to that in other studies with a different definition of restenosis.^9^ However, this result does not indicate that EBD is an ineffective therapy. The study assessed the effect of only one session of dilation and found a significant increase in the diameters of strictures. This result suggests that repeating scheduled DBE with maintenance EBD has the potential to decrease stricture numbers and avoid the need for surgical intervention.

To evaluate long‐term outcomes after EBD, symptom‐free remission rate and surgery‐free rate were usually assessed in previous studies.^10^, ^11^, ^12^ However, in practice, eating habits and adherence to elemental diet therapy in patients can affect the presence of obstructive symptoms. The surgery‐free rate might depend on the intentions of the patient and the treating physician. Therefore, risk factors for restenosis are not defined yet. Hibiya et al. reported that the presence of an ulcer at the site of stricture is a possible risk factor for needing repeat dilation and surgical resection after EBD in a study of 98 patients. Patients with ulcers at a stricture had a significantly higher risk of requiring surgery than those with mucosal healing (hazard ratio 4.84, 95% confidence interval 1.58–14.79).^21^ In the current study, even small and shallow ulcerations affected the outcomes after EBD. Results show that the maximum improvement of the narrowest stricture (+1.7 mm) was observed among patients in the no‐ulcer group, who had complete mucosal healing seen at both sessions. Conversely, the other three groups, who had small ulcerations at either any or both sessions, did not show significant improvement.

EBD up to 15 mm was negatively associated with restenosis. This suggests that EBD for moderate to mild strictures is more effective than EBD for severe strictures and supports the advantage of scheduled maintenance EBD.

The presence of small ulceration at the follow‐up session was identified as a risk factor for restenosis based on multivariate analysis in the present study. These results suggest that maintaining complete mucosal healing is important to improve the long‐term outcomes of EBD.

A systematic review by Schulberg et al. concluded that effective anti‐inflammatory drug therapy combined with optimal endoscopic therapy for strictures provides the most effective non‐surgical treatment of strictures.^22^ However, clinical decision‐making to prevent restenosis was often difficult in patients with small ulcers with clinical and serological remission. The results of the present study have made it more straightforward. Complete mucosal healing should be achieved and maintained by post‐EBD treatment optimization based on endoscopic findings as the treat‐to‐target strategy.

This study has several limitations. First, it is a single‐center retrospective study with a relatively small number of patients. Therefore, the influence of the choice of medical treatment could not be evaluated. Secondly, it is possible that the comparison may not be of exactly the same stricture, although comparing the diameters of strictures one‐to‐one was ideal. Most patients had multiple strictures, and some mild strictures became unrecognizable at the follow‐up session. Comparing them one‐to‐one becomes very complicated. The narrowest stricture in each patient is often responsible for symptoms and rarely changes at the two sessions. For these reasons, we considered it better to compare the narrowest stricture for this analysis.

In conclusion, maintenance of complete mucosal healing is significantly associated with avoiding restenosis after EBD for CD‐related small intestinal strictures.

## CONFLICT OF INTEREST STATEMENT

Hironori Yamamoto has patents for the calibrated small‐caliber‐tip transparent hood. Hironori Yamamoto has patents for double‐balloon endoscopy and a consultant relationship with Fujifilm. Tomonori Yano and Hirotsugu Sakamoto have received research funding and honoraria from Fujifilm.

No other authors have personal financial relationships with a commercial entity producing healthcare‐related products and/or services relevant to this article.
